# The Effect of Traumatic Brain Injury on the Gastrointestinal System: A Comprehensive Review

**DOI:** 10.3390/brainsci16030254

**Published:** 2026-02-25

**Authors:** Ruhi K. Shah, Justin J. Lin, Tejaswi Makkapati, Arielle A. Berkowitz, Brian D. Greenwald

**Affiliations:** 1 Hackensack Meridian School of Medicine, Nutley, NJ 07110, USA; 2 Department of Physical Medicine and Rehabilitation, JFK Johnson Rehabilitation Institute at Hackensack Meridian Health, Edison, NJ 08820, USA

**Keywords:** traumatic brain injury, autonomic dysregulation, gut–brain axis, gastrointestinal dysregulation

## Abstract

**Highlights:**

**What are the main findings?**

- Traumatic brain injury can lead to widespread gastrointestinal dysfunction through disruptions of the brain–gut axis, dysmotility, dysbiosis, malnutrition, and metabolic disturbances.
- Patients with TBI may experience GI disturbances, including dysphagia, gastroparesis, and sialorrhea, among many others.

**What are the implications of the main findings?**

- Routine assessment and early management of gastrointestinal dysfunction should be integrated into standard TBI care to optimize recovery.
- Targeted interventions, such as early enteral nutrition, represent promising strategies to improve neurorecovery.

**Abstract:**

Background/Objectives: Traumatic brain injury (TBI) is a significant public health concern resulting in physical, cognitive, and behavioral impairments. Emerging evidence highlights a bidirectional relationship between brain injury and gut health, known as the brain–gut axis. This paper provides a comprehensive review of current literature exploring the relationship between TBI and various gastrointestinal (GI) pathologies, examining how brain injuries contribute to GI dysfunction and how gut health influences neurorecovery. Methods: A comprehensive search of peer-reviewed articles was conducted between March and June 2025 using databases including PubMed, Scopus, and Cochrane. Studies from 2010 onwards involving human subjects were screened. Search terms included combinations of “traumatic brain injury,” “TBI,” and “[gastrointestinal pathology].” Data regarding study design, population, GI outcomes, and proposed mechanisms were analyzed. Results: TBI triggers secondary injury cascades, including neuroinflammation, dysautonomia, and gut microbiome dysbiosis. The review identifies a wide spectrum of TBI-associated GI disorders, including dysphagia, esophageal disorders, gastric disorders, and intestinal disorders. Bowel dysfunction, manifesting as constipation or incontinence, is prevalent due to neurogenic factors and cognitive impairments. Additionally, metabolic dysregulation following TBI leads to malnutrition, hyperglycemia, and hypoglycemia, all of which impact morbidity. Conclusions: The GI system is integrally connected to TBI recovery through immune modulation and nutrient absorption. Dysfunction within the brain–gut axis, specifically altered motility, permeability, and inflammation, contributes to secondary brain injury and impedes neurological outcomes. Clinical assessment of GI dysfunction should be integrated into routine TBI care. Therapeutic strategies, including early enteral nutrition, are essential to optimize recovery and reduce systemic inflammation.

## 1. Introduction

Traumatic brain injury (TBI) is a significant public health concern that can result in a wide spectrum of physical, cognitive, and behavioral impairments [1]. TBIs occur when there is an external force to the brain caused by an impact to the head and/or body. The most common causes of TBI include falls, especially in older adults, as well as motor vehicle accidents and sports injuries, which tend to affect younger individuals [2].

The effects of TBI can range from mild and temporary to severe and permanent, depending on the nature and severity of the injury. Recovery from TBI is unique to each individual and may be impacted by TBI severity. Following TBI, many patients require long-term rehabilitation and support and are often at greater risk for a host of medical complications [3].

One often overlooked consequence of TBI is its impact on gastrointestinal (GI) function. Emerging evidence suggests a bidirectional relationship between brain injury and gut health [4]. The initial TBI triggers a secondary injury cascade characterized by glutamate excitotoxicity, intracellular calcium overload, mitochondrial dysfunction, and oxidative stress, which can lead to neuronal cell loss, synaptic failure, and cognitive deficits. This secondary injury is propagated by chronic neuroinflammation, driven by the sustained activation of microglia and astrocytes.

Bacterial extracellular vesicle dysfunction has been implicated as an additional mediator of GI pathology in patients with traumatic brain injury. These vesicles influence neuroinflammation, neuronal survival, and immune responses and have the ability to cross the blood–brain barrier [5]. In response to gut dysbiosis following TBI, imbalanced bacterial endosome production can exacerbate inflammation and metabolic dysregulation, creating pathological feedback loops that aggravate both the central nervous system and the GI system [6,7].

Arousal is disrupted through direct damage to key regulatory centers in the brainstem and hypothalamus, leading to hypothalamic–pituitary–adrenal (HPA) axis dysregulation and a “sympathetic storm” that overwhelms the parasympathetic system [8].

Distinct and sustained changes occur in the gut microbiome, with downstream effects on the gut–brain axis that may worsen neurological outcomes. Animal models demonstrate that TBI triggers gut microbial dysbiosis, impaired intestinal barrier function (leading to increased permeability and bacterial translocation), and altered microbial metabolite profiles that correspond with persistent microglial activation, neuroinflammation, and worse recovery [9]. Additionally, post-TBI, many patients are treated with medications that exacerbate the gut–brain dysfunction; these include antibiotics, nonsteroidal anti-inflammatory drugs (NSAIDs), and proton pump inhibitors (PPIs), which drastically alter gut microbiota composition [10,11]. Longitudinal studies find that the post-injury microbiome remains significantly altered in composition months after injury [12]. TBI-associated gut dysbiosis is thought to contribute to the increased susceptibility of TBI patients to neurodegenerative processes, such as Parkinson’s disease [13]. These findings have prompted consideration of microbiome-targeted interventions (probiotics, prebiotics, and fecal microbiota transplantation) as potential adjuvants in patients recovering from brain injuries.

The disruption of the gut–brain axis and the autonomic nervous system can contribute to a wide range of GI issues, including dysphagia, dysmotility, and constipation [4,14]. In addition, altered arousal resulting from TBI, including hypersomnia, fatigue, and disorders of consciousness, can impact patients’ ability to feed themselves, in turn, causing poor oral intake and malnutrition [15]. This highlights the importance of understanding how gut health can influence neurorecovery, or conversely, how brain injuries contribute to GI dysfunction. The purpose of this paper is to provide a comprehensive review of the current literature exploring the relationship between TBI and various GI pathologies.

## 2. Materials and Methods

### 2.1. Search Strategy

A comprehensive search was conducted to identify peer-reviewed articles examining the relationship between TBI and GI dysfunction. Multiple databases were analyzed in order to conduct this review: PubMed, Scopus, and Cochrane. The search was performed between March and June 2025. Search terms included combinations of keywords and MeSH terms such as “traumatic brain injury” OR “TBI” AND “gastrointestinal” OR “gut”. Further search terms can be found in Appendix A. Boolean operators (AND, OR) and filters were used to refine the search results.

Relevant articles and reviews were manually screened to identify additional studies. All identified articles were screened for relevance by two independent reviewers based on titles and abstracts. Full-text reviews were conducted for articles that met the inclusion criteria or when eligibility was unclear. Discrepancies were resolved through discussion or consultation with a third reviewer.

Key data extracted included study design, population characteristics, type and severity of TBI, GI outcomes assessed, underlying mechanisms proposed (e.g., microbiome alterations, vagus nerve signaling, intestinal permeability), and major findings.

### 2.2. Inclusion and Exclusion Criteria

Studies were included if they met the following criteria:

- Published in English;
- Involved human subjects;
- Addressed GI outcomes, mechanisms, or dysfunctions associated with TBI;
- Peer-reviewed original research, systematic reviews, or meta-analyses;
- Articles published from 2010 onwards, aside from key references found in the literature.

Exclusion criteria included:

- Conference abstracts, editorials, and opinion pieces;
- Studies not pertaining to TBI;
- Articles not available in full text.

## 3. Results

### 3.1. Dietary Considerations (Table 1)

#### 3.1.1. Cognition, Arousal, Olfaction, and Ageusia

Cognitive impairment and arousal disturbances following a TBI can impact nutritional intake. Damage to the frontal lobe can impact executive function and, therefore, impact the ability to feed oneself and maintain proper nutrition. Executive functioning, which includes planning, organization, task initiation, and working memory, renders multi-step activities like meal preparation overwhelmingly difficult, often leading to task avoidance, reliance on nutrient-poor processed foods, or skipped meals entirely [16,17,18]. Memory impairments further compound this issue, as anterograde amnesia can cause individuals to forget to eat, while disinhibition and poor impulse control linked to orbitofrontal cortex damage can manifest as hyperphagia and an uncontrollable craving for highly palatable foods [19,20,21,22]. Disturbances in smell due to olfactory nerve injury and frontal lobe damage can also impact appetite [23,24]. Concurrently, disturbances in arousal, including sleep–wake cycle disruption, can act as chronic stressors that promote gut microbiome dysbiosis and compromise the integrity of the intestinal barrier [25,26,27,28].

**Table 1:** **Dysphagia,** Hyper-, and hypoglycemia.

Disorder	Mechanisms Post-TBI	Symptoms	Treatment
Dysphagia	Disruption of neural pathways controlling the swallowing muscles	Difficulty swallowing Coughing or choking Sensation of food being stuck Recurrent pneumonia	Swallowing rehabilitation therapy Dietary modifications (texture/consistency) Neuromuscular electrical stimulation Pharyngeal electrical stimulation
Hyperglycemia	Stress-induced physiological response to severe injury	Polyuria, polydipsia Dehydration Altered mental status	Insulin therapy Conventional glycemic control is often preferred Nutritional support
Hypoglycemia	Often, a complication of intensive insulin therapy used to treat hyperglycemia	Confusion, altered mental status Seizures, diaphoresis Tachycardia, loss of consciousness	Prompt IV glucose administration Continuous glucose monitoring Adjustment of insulin therapy Adequate nutritional support

#### 3.1.2. Malnutrition

For patients with TBI, nutritional support is crucial, as managing glucose levels alongside providing necessary calories and proteins, preferably via enteral nutrition, is needed to support recovery and meet a patient’s metabolic needs [29,30].

Malnutrition is seen in roughly 60% of hospitalized patients with moderate-to-severe TBI. Pulmonary infection, urinary tract infection, application of nasogastric tubes, low Glasgow Coma Scale (GCS) scores, and low Activities of Daily Living (ADL) scores may contribute to malnutrition [31].

It is estimated that 25–30 percent of patients with TBI also have dysphagia, which can cause coughing or choking with food intake as well as the sensation of food being “stuck in the throat,” termed globus sensation [32]. This can worsen malnutrition as well as result in dehydration and aspiration pneumonia [33,34]. Its prevalence is higher in individuals with TBI because the injury can disrupt the neural pathways that control the swallowing muscles. Treatment includes swallowing rehabilitation therapy, dietary modifications, and neuromuscular electrical stimulation to improve muscle function [34,35,36].

Patients with TBI experience considerable energy and protein deficits, which persist throughout their hospitalization, including both ICU and ward-based care [37]. These deficits are associated with adverse outcomes, including increased morbidity and mortality [37,38]. Symptoms of malnutrition in patients with TBI include weight loss, muscle wasting, impaired wound healing, increased susceptibility to infections, and prolonged recovery times. These symptoms are due to the hypermetabolic and hypercatabolic states induced by TBI, which increase nutritional requirements [39,40].

Treatment options for malnutrition in TBI patients focus on early and adequate nutritional support. Enteral nutrition is preferred and should be initiated as soon as the patient is hemodynamically stable, ideally within 72 h of injury, to reduce infection rates and complications [41]. More specifically, studies have demonstrated that early enteral nutrition in patients with TBI has led to outcomes such as significantly improved GCS scores; significantly lower decreases in tools of nutritional assessment, including serum albumin and protein and mid-arm muscle circumference; and significantly improved hormonal profiles, including significantly lower declines in TSH, free T3 and T4, and testosterone (in males) and significantly lower rises in cortisol [42,43,44]. The goal of early enteral feeding is to provide 25–35 non-protein kcal/kg/day and 2.0–2.5 g protein/kg/day. If enteral feeding is not feasible, parenteral nutrition should be considered [40]. Immunonutrition, which includes nutrients like omega-3 fatty acids and curcumin, may also help reduce inflammation and improve outcomes [45,46].

Monitoring nutritional status using indirect calorimetry and nitrogen balance studies is recommended to tailor nutritional interventions and prevent complications such as hyperglycemia [29]. This comprehensive approach aims to optimize recovery and improve clinical outcomes in TBI patients.

#### 3.1.3. Hyperglycemia and Hypoglycemia

Hyperglycemia is a common occurrence in patients suffering from TBI. The prevalence of hyperglycemia in this patient population varies across studies, but it is generally reported to be significant. A study by Matovu et al. (2021) found that nearly one in six patients with severe TBI were admitted with hyperglycemia, defined as blood glucose levels greater than 11.1 mmol/L (200 mg/dL) [47]. Another study by El-Menyar et al. (2021) reported that 13% of trauma patients presented with hyperglycemia upon admission, with half of these cases being stress-induced hyperglycemia (SIH) [47,48]. This is significant because SIH is associated with poor neurological outcomes and higher morbidity and mortality rates in TBI patients [49,50,51,52]. Additionally, many TBI patients may be treated with steroids, which can also contribute to hyperglycemia [53]. Symptoms of hyperglycemia in patients with TBI include polyuria, polydipsia, dehydration, altered mental status, and, in severe cases, diabetic ketoacidosis or hyperosmolar hyperglycemic state.

The prevalence of hypoglycemia in patients suffering from TBI is notably influenced by the glycemic control strategy employed. According to a systematic review and meta-analysis by Hermanides et al., intensive glycemic control in TBI patients significantly increases the risk of hypoglycemia compared to conventional control [54]. The study found that severe hypoglycemia occurred more frequently with intensive glucose control compared to a relative risk of 0.22 (95% CI: 0.09–0.52) for conventional control, indicating a lower incidence of hypoglycemia in this group. The NICE-SUGAR study subgroup analysis by Finfer et al. also reported that moderate hypoglycemia (blood glucose 2.3–3.9 mmol/L) occurred in 79.2% of patients under intensive control, compared to 9.0% under conventional control [55]. Furthermore, severe hypoglycemia (blood glucose ≤ 2.2 mmol/L) was observed in 4.9% of patients with intensive control and none with conventional control. Thus, with intensive glycemic control strategies, the prevalence of hypoglycemia in TBI patients is significantly increased, underscoring the need for careful monitoring and management of blood glucose levels in this population. Symptoms of hypoglycemia include confusion, altered mental status, seizures, diaphoresis, tachycardia, and, in severe cases, loss of consciousness, which can exacerbate the neurological deficits already present and complicate the clinical management of these patients.

### 3.2. Disorders of the Oral Cavity (Table 2)

#### 3.2.1. Oral Ulcers

Oral ulcers are small, painful lesions that form on the mucous membranes inside the mouth, causing discomfort while eating and/or speaking. Patients with TBI have higher rates of dental plaque, gingival inflammation, and oral infections [56,57]. Additionally, they face changes in nutrition, and the combination of these factors may contribute to the development of oral ulcers [58]. Treatments include maintaining good oral hygiene, using topical corticosteroids to reduce inflammation, and applying protective pastes to cover the lesions. In severe cases, systemic options like oral corticosteroids or immunosuppressants may be considered [59].

**Table 2:** Disorders of the oral cavity.

Disorder	Mechanisms Post-TBI	Symptoms	Treatment
Oral Ulcers	Immune dysregulation Increased stress Altered oral hygiene and nutritional intake	Small, painful lesions in the mouth Burning sensation Difficulty eating and speaking	Good oral hygiene Topical Corticosteroids Protective pastes Systemic corticosteroids/immunosuppressants
Oral Candidiasis (Thrush)	Immune dysregulation/suppression Prolonged hospitalization and antibiotic use Poor oral hygiene	White, creamy lesions on tongue/cheeks Pain and redness Difficulty swallowing Cracking at mouth corners	Emphasis on good oral hygiene Topical antifungals (e.g., nystatin) Systemic antifungals (e.g., fluconazole) for severe cases Emphasis on good oral hygiene
Herpes Simplex Virus (HSV) Reactivation	Systemic immunosuppression following injury allows the latent virus to reactivate	Painful vesicular lesions (cold sores) Fever, headache Severe cases: encephalitis, seizures	Antiviral medications (e.g., acyclovir, valacyclovir)
Sialorrhea	Impaired neuromuscular control of orofacial muscles, affecting saliva management and swallowing	Excessive, unintentional loss of saliva from the mouth Can lead to skin irritation and aspiration	Anticholinergic medications (e.g., glycopyrrolate) Botulinum toxin injections into salivary glands Radiation therapy

#### 3.2.2. Oral Candidiasis

Oral candidiasis, or thrush, is a fungal infection caused by *Candida* species that creates painful, white lesions in the mouth, often accompanied by a cottony feeling and loss of taste. The prevalence of oral candidiasis is increased in TBI patients, likely due to an immunosuppressive state following the injury, prolonged hospitalization with broad-spectrum antibiotic use that disrupts oral flora, salivary gland hypofunction, and poor oral hygiene resulting from physical or cognitive impairments [60,61,62]. Treatment guidelines recommend topical antifungals such as nystatin or clotrimazole as first-line therapy for mild cases, with systemic antifungals such as fluconazole reserved for moderate-to-severe cases, alongside an emphasis on good oral hygiene [63].

#### 3.2.3. Herpes Simplex Virus

Herpes simplex virus (HSV) infection can cause conditions such as oral herpes, characterized by painful blisters or sores on the lips, mouth, and/or gums. The virus establishes latency in sensory neurons and may reactivate during periods of stress/immunosuppression, as seen following TBI [61,64,65,66,67]. One study noted the reactivation of HSV infection in 39% of TBI patients who required mechanical ventilation [65]. Treatment guidelines recommend intravenous acyclovir for severe disease or encephalitis, with oral antivirals like valacyclovir and famciclovir for less severe cases, and foscarnet or cidofovir for acyclovir-resistant infections [68,69].

#### 3.2.4. Sialorrhea

Sialorrhea, or excessive drooling, is the unintentional loss of saliva from the mouth, which can lead to skin irritation and an increased risk of aspiration pneumonia [70,71]. The prevalence of sialorrhea is increased in individuals with TBI due to impaired neuromuscular control of the orofacial muscles, which affects the ability to manage saliva effectively [72]. First-line pharmacologic therapy includes anticholinergic medications such as glycopyrrolate or scopolamine patches to reduce saliva production [70,73]. If these are not effective, botulinum toxin injections into the salivary glands are recommended, and in refractory cases, radiation therapy may be considered [73,74].

### 3.3. Esophageal Disorders (See Table 3)

#### GERD, Esophagitis, and Barrett’s Esophagus

Gastroesophageal reflux disease (GERD) is a chronic condition where stomach contents flow back into the esophagus, causing symptoms that include heartburn, regurgitation, chest pain, and extraesophageal manifestations such as chronic cough and laryngitis. The prevalence of GERD is increased in TBI patients due to systemic inflammation and impaired autonomic nervous system function, which can decrease lower esophageal sphincter tone and delay gastric emptying [58,75,76,77,78]. Additionally, TBI patients with altered mobility may be on medications that exacerbate GERD symptoms. Guidelines for treatment include lifestyle modifications such as weight loss, avoiding meals close to bedtime, eliminating spicy/acidic foods, and elevating the head of the bed [79]. The primary pharmacologic treatment is proton pump inhibitors [79,80,81]. Complications include progression to Barrett’s esophagus, esophagitis, and esophageal carcinoma.

**Table 3:** Esophageal disorders.

Disorder	Mechanisms Post-TBI	Symptoms	Treatment
GERD	Impaired autonomic nervous system function Decreased lower esophageal sphincter tone Delayed gastric emptying	Heartburn Regurgitation Chest pain Chronic cough, laryngitis	Weight loss Avoiding late meals, trigger foods, to-bacco Elevating head of bed Proton pump inhibitors (PPIs)

### 3.4. Gastric Disorders (Table 4)

#### 3.4.1. Gastritis, Peptic Ulcer Disease, and Cushing Ulcer

Gastritis is an inflammation of the stomach lining that can cause symptoms such as epigastric pain, nausea, vomiting, bloating, loss of appetite, and, in severe cases, hematemesis or melena. The prevalence of gastritis is higher in individuals with TBI due to factors such as dysautonomia, which impairs gastric protection, and the common use of medications such as NSAIDs and corticosteroids that can damage the stomach lining [75,76,82,83,84]. TBI-induced dysautonomia can lead to impaired gastric motility and reduced mucosal defense. This mechanism is also implicated in the increase in the prevalence of peptic ulcers in patients with TBI.

**Table 4:** Gastric disorders.

Disorder	Mechanisms Post-TBI	Symptoms	Treatment
Gastritis	Dysautonomia affecting gastric motility and mucosal protection Use of NSAIDs and corticosteroids	Epigastric pain, nausea, vomiting Bloating, loss of appetite Hematemesis or melena	Proton pump inhibitors (PPIs) H2-receptor antagonists (H2RAs) Avoidance of NSAIDs
Peptic Ulcer Disease and Cushing Ulcers	Increased gastric acid secretion from stress (Cushing ulcers) Use of NSAIDs and corticosteroids	Epigastric pain (burning/gnawing) Nausea, vomiting, bloating Hematemesis or melena	Proton pump inhibitors (PPIs) H2-receptor antagonists (H2RAs) Avoidance of NSAIDs
Gastroparesis and Recurrent Emesis	Dysautonomia impairing gastric motility Neuroinflammation and systemic inflammation	Nausea, vomiting Early satiety, bloating Postprandial fullness, abdominal pain	Dietary modifications (low-fat, low-fiber) Prokinetic and antiemetic agents Gastric electrical stimulation Hydration and nutritional support

In patients with brain injury, peptic ulcers are specifically referred to as Cushing ulcers [85]. These ulcers occur in the lining of the stomach or the first part of the small intestine, causing burning or gnawing epigastric pain, nausea, vomiting, bloating, and, in severe cases, hematemesis or melena [85,86]. In addition to dysautonomia and neuroinflammation, it is also thought that elevated intracranial pressure stimulates the vagus nerve, leading to hypersecretion of gastric acid and subsequent ulcer formation due to stress-related mucosal damage [76,83,87,88]. Treatment of these conditions focuses on reducing stomach acid with PPIs or H2-receptor antagonists, limiting NSAIDs and corticosteroids, eliminating *H. pylori* infection if present, and lifestyle changes such as avoiding alcohol and smoking [89]. For bleeding ulcers, endoscopic therapy is used to stop the bleeding, and sucralfate may be used as an adjunctive therapy to protect the stomach lining [89,90,91].

#### 3.4.2. Gastroparesis

Gastroparesis is a disorder characterized by delayed emptying of the stomach in the absence of a blockage, causing symptoms like nausea, vomiting, early satiety, bloating, postprandial fullness, and abdominal pain. Its prevalence is estimated to be 45–50% in TBI patients due to dysautonomia, which impairs the autonomic nervous system’s control over gastric motility [76,92]. In addition to TBI-associated neuroinflammation, medications such as opioids can also contribute to this delay in stomach emptying [78,93]. Treatment includes dietary modifications, such as small, frequent, low-fat, and low-fiber meals. Prokinetic agents, such as metoclopramide, are used to improve stomach emptying, and antiemetic agents can help control nausea, and for severe cases, gastric electrical stimulation or a gastric peroral endoscopic myotomy may be considered [94,95].

### 3.5. Disorders of the Intestines (Table 5)

#### 3.5.1. Duodenitis

Duodenitis is an inflammation of the duodenum, which can be caused by various factors, including infections (e.g., *Helicobacter pylori*), NSAIDs, and autoimmune diseases. It can be characterized by upper abdominal pain, nausea, vomiting, bloating, and loss of appetite, as well as GI bleeding in severe cases. While its prevalence in TBI patients is not explicitly quantified, there is substantial evidence that TBI-induced neuroinflammation and dysautonomia can disrupt the brain–gut axis and alter gut microbiota and bile acid profiles, leading to increased intestinal permeability and inflammation, both of which are factors that can contribute to duodenitis [58,75,83]. Management of duodenitis typically involves addressing the underlying cause, such as eradicating *H. pylori* infection with antibiotics or discontinuing NSAIDs. PPIs are commonly used to reduce gastric acid secretion and promote mucosal healing [96].

**Table 5:** Disorders of the intestines.

Disorder	Mechanisms Post-TBI	Symptoms	Treatment
Duodenitis	Neuroinflammation and dysautonomia Increased intestinal permeability Alterations in gut microbiota	Upper abdominal pain Nausea, vomiting Bloating, loss of appetite	Address underlying cause (e.g., *H. pylori*) Proton Pump Inhibitors
Small Intestinal Bacterial Overgrowth (SIBO)	Gut microbiota dysbiosis Increased intestinal permeability Systemic inflammation and dysautonomia Changes in gut motility	Abdominal pain, bloating, gas, distension Diarrhea or constipation Nausea, cramping	Antibiotics (e.g., Rifaximin) Dietary modifications Probiotics and prokinetic agents
Small Bowel Obstruction	Dysmotility from systemic and neuroinflammation Increased intestinal permeability	Severe, crampy abdominal pain Nausea and bilious vomiting Abdominal distension Inability to pass stool or gas	Nasogastric decompression IV fluids Prokinetic agents Surgical intervention if needed
Superior Mesenteric Artery (SMA) Syndrome	Predisposing factors like significant weight loss and prolonged immobilization can occur post-TBI	Severe postprandial epigastric pain Nausea and bilious vomiting Early satiety, weight loss	Nutritional support Positional therapy Surgical intervention
Constipation/Incontinence	Disruption of the brain–gut axis -Neuroinflammation Dysautonomia	Fewer than three bowel movements per week Hard stools, straining, bloating Feeling of incomplete evacuation	Increased dietary fiber Osmotic agents Stimulant laxatives Biofeedback therapy
Irritable Bowel Syndrome	Neuroinflammation and systemic inflammation Dysmotility and increased mucosal permeability Gut microbiota dysbiosis	Recurrent abdominal pain Bloating Diarrhea, constipation, or alternating	Dietary modifications Antispasmodics Probiotics
Ischemic Colitis	Dysautonomia, systemic inflammation, and increased intestinal permeability can exacerbate the condition	Sudden onset of abdominal pain Rectal bleeding Diarrhea	Supportive care including bowel rest, IV fluids Antibiotics Surgical intervention
Proctitis	Systemic inflammation and dysautonomia may predispose patients to proctitis	Rectal pain, tenesmus Rectal bleeding and discharge Urinary urgency	Mesalamine Oral 5-ASA agents Systemic therapies (refractory cases)
Clostridium difficile and Sequelae	Gut dysbiosis and increased mucosal permeability can predispose to *C. difficile* overgrowth	Watery diarrhea Abdominal pain and cramping Fever, nausea, dehydration	Discontinue inciting antibiotic Oral vancomycin or fidaxomicin Hydration and electrolyte management

#### 3.5.2. Small Intestinal Bacterial Overgrowth

Small intestinal bacterial overgrowth (SIBO) is a clinical syndrome characterized by the presence of an excessive number of bacteria in the small intestine, defined by a bacterial colony count of ≥10^3^ colony-forming units per milliliter (CFU/mL) in a duodenal or jejunal aspirate [97]. Symptoms commonly include abdominal pain, bloating, gas, distension, flatulence, and diarrhea, which are prevalent in more than two-thirds of patients [97]. While the prevalence of SIBO in patients with TBI is not explicitly quantified, TBI has been shown to cause gut microbiota dysbiosis, increased intestinal permeability, systemic inflammation, dysautonomia, and changes in gut motility, all of which can contribute to the development of SIBO [58,75,83,98]. Treatment of SIBO primarily involves the use of antibiotics to reduce the bacterial load, such as rifaximin (first-line), metronidazole, ciprofloxacin, and amoxicillin–clavulanate [99]. Adjunctive therapies include dietary modifications, probiotics, and prokinetic agents to enhance intestinal motility and prevent recurrence [99].

#### 3.5.3. Small Bowel Obstruction and Paralytic Ileus

Small bowel obstruction (SBO) occurs when there is a blockage within the intestinal tract that prevents food, liquid, gas, and stool from passing normally through the bowel, while paralytic ileus is a condition characterized by the absence of intestinal peristalsis without any mechanical obstruction. Both conditions share common symptoms, which typically involve severe, crampy abdominal pain, nausea and bilious vomiting, abdominal distension, absence of bowel sounds, and eventually, inability to pass gas or stool [75,76,100,101]. The prevalence of SBO and paralytic ileus in patients with TBI is limited. However, TBI is known to cause significant gastrointestinal dysfunction, including dysmotility and increased intestinal permeability, as well as neuroinflammation and dysautonomia, all of which can predispose to both SBO and paralytic ileus [75,76].

Treatment for both conditions begins with nasogastric decompression and nothing by mouth (NPO) status for patients to relieve bowel distension and vomiting, as well as IV fluids for hydration and electrolyte balance. Prokinetic agents, such as metoclopramide, which can be utilized to stimulate bowel motility, may also be given but must be considered carefully in the context of TBI-induced dysautonomia. Lastly, encouragement of early ambulation can be vital to care, as it has been shown to stimulate bowel function [75,76,101].

#### 3.5.4. Superior Mesenteric Artery Syndrome

Superior mesenteric artery syndrome (SMAS) is a rare condition characterized by compression of the third portion of the duodenum between the aorta and the superior mesenteric artery (SMA), leading to duodenal obstruction and symptoms of severe postprandial epigastric pain, nausea and vomiting (frequently bilious), early satiety, weight loss, and abdominal distension and bleeding [102,103,104]. Although specific quantitative data on the prevalence of SMAS in TBI patients are lacking, systemic inflammation, dysautonomia, and prolonged immobilization can contribute to gastrointestinal complications and significant weight loss, predisposing TBI patients to SMAS via a reduction in the angle between the aorta and the SMA. To diagnose SMAS, physicians must identify its characteristic clinical symptoms along with CT or CT angiography showing the presence of a reduced aortomesenteric angle (≤22°) and aortomesenteric distance (≤8 mm), with proximal duodenal and gastric dilation and narrowing of the third portion of the duodenum [105]. Treatment guidelines recommend conservative management first, such as enteral feeding via a nasojejunal tube or total parenteral nutrition, to promote weight gain and relieve duodenal compression. Positional therapy to relieve compression and metoclopramide to enhance GI motility may also be utilized. In severe cases, a duodenojejunostomy can be pursued to bypass the compressed segment [104,106].

#### 3.5.5. Constipation/Incontinence

Bowel incontinence is the involuntary loss of solid or liquid stool, typically presenting as unintentional soiling, unawareness of the need to defecate, or inability to defer defecation [107]. In patients with TBI, the prevalence of bowel incontinence was highest during the acute phase of TBI rehabilitation, with 68% of patients experiencing incontinence at admission to inpatient rehabilitation, according to a retrospective study conducted by Foxx-Orenstein et al. [108]. This prevalence decreased to 12.4% at discharge and 5.2% at one-year follow-up, highlighting the acute nature of bowel incontinence [108]. One reason for bowel incontinence in TBI stems from cognitive deficits, such as impaired awareness, attention, executive function, and memory, which can reduce patients’ ability to recognize the need to defecate, respond appropriately to rectal sensations, or perform timely toileting behaviors. Moreover, TBI-induced behavioral disturbances, such as apathy, disinhibition, and lack of motivation, can cause incontinence by interfering with adherence to bowel routines or the initiation of toileting. Lastly, TBI patients who simply have reduced mobility or loss of ability to control their external anal sphincter can also suffer from bowel incontinence, as well as incontinence-associated dermatitis, which can stem from inadequate cleansing, prolonged contact with moisture, and prolonged pressure on the skin for these TBI patients with limited mobility [108,109]. First-line management of bowel incontinence is typically a structured bowel program with scheduled toileting, dietary fiber optimization, environmental modifications (e.g., use of commodes), and use of stool-bulking agents alongside antidiarrheal medications to both improve stool consistency and reduce urgency [110,111]. For patients with partial neurological injury, a treatment plan that combines sensory retraining with pelvic floor muscle exercises can be utilized [112]. Finally, transanal irrigation, sacral neuromodulation, and surgical interventions can be indicated as options for symptoms refractory to conservative measures and less invasive therapies [110,112,113,114].

Constipation is defined as having fewer than three bowel movements per week, often accompanied by symptoms such as hard stools, a feeling of incomplete evacuation, abdominal discomfort, bloating, excessive straining, and a sense of anorectal blockage during defecation [115,116]. While the prevalence of constipation in patients with TBI is not precisely defined within the literature, it is a commonly observed complication that can stem from disruption of the brain–gut axis, neuroinflammation, and dysautonomia, as shown by studies indicating that bowel dysfunction, including constipation, affects nearly two out of three patients with acquired brain injury [75,111]. Increasing dietary fiber intake is recommended as a first-line treatment and can be achieved through fiber-rich foods or supplements [115]. Pharmacological treatments, including osmotic agents such as polyethylene glycol or milk of magnesia, are also commonly used, as well as stimulant laxatives like bisacodyl or glycerol suppositories [115]. Lastly, for refractory cases, newer agents such as lubiprostone and linaclotide may be considered, and in severe cases, colonic manometry and barostat testing may be required to evaluate colonic motility [115].

#### 3.5.6. Irritable Bowel Syndrome

Irritable bowel syndrome (IBS) is a chronic disorder of gut–brain interaction characterized by recurrent abdominal pain associated with changes in stool frequency or form [117,118]. The prevalence of IBS in patients with TBI is not explicitly detailed, although studies have shown that TBI can induce gut microbiota dysbiosis and intestinal inflammation, both of which are factors implicated in the pathogenesis of IBS [75,83]. Additionally, TBI is known to cause significant neuroinflammation and systemic inflammation, which can lead to conditions such as dysmotility and increased mucosal permeability, which overlap with the symptoms present in IBS [75,76,83]. Treatment options for IBS first include dietary modifications, with the American Gastroenterological Association (AGA) recommending a low-FODMAP diet [119]. Second-line treatments include antispasmodics for pain, antidiarrheals for diarrhea, and laxatives for constipation, as well as intestinal secretagogues, central neuromodulators, and drugs acting on opioid or 5-HT receptors in more severe cases of IBS [117,118]. Finally, cognitive–behavioral therapy and mindfulness treatments have also shown efficacy in improving symptoms and quality of life [119,120].

#### 3.5.7. Mesenteric Ischemia, Ischemic Colitis, and Proctitis

Mesenteric ischemia is a condition characterized by reduced blood flow to the intestines, leading to ischemic injury, which can lead to symptoms of severe abdominal pain out of proportion to physical findings, nausea and vomiting, diarrhea or bloody stools, abdominal distension, and fever and leukocytosis, which can indicate possible bowel necrosis [75,76,121]. Ischemic colitis is a condition characterized by reduced blood flow to the colon, which can lead to symptoms of sudden onset of abdominal pain, often associated with tenderness, rectal bleeding, diarrhea, and fever [122]. Proctitis is inflammation of the rectum, typically the distal 10–12 cm, which can lead to symptoms of rectal pain, tenesmus, rectal bleeding, discharge, urgency, and lower abdominal pain [123]. There are no specific data on the prevalence of mesenteric ischemia, ischemic colitis, or proctitis in patients with TBI in the medical literature. However, TBI can lead to gastrointestinal dysfunction through mechanisms involving the brain–gut axis, systemic inflammation, and dysautonomia, all of which may predispose patients to various GI conditions, including mesenteric ischemia, ischemic colitis, or proctitis, via endothelial dysfunction, impaired vasoconstriction/vasodilation of arteries, and impaired blood flow [75,76,121,124].

Despite potentially similar pathophysiological mechanisms, the treatment of these three conditions differs significantly. For mesenteric ischemia, resuscitation with IV fluids, hemodynamic support, and broad-spectrum antibiotics constitute first-line procedures to prevent sepsis. After patient stabilization, angioplasty and stenting can be performed to revascularize occluded mesenteric arteries, with surgical interventions such as bypass performed in cases where endovascular treatment is not feasible [125]. For ischemic colitis, the American Gastroenterological Association (AGA) emphasizes the importance of early diagnosis and appropriate supportive care with bowel rest, IV fluids, and antibiotics to prevent secondary infection. In severe cases, surgical intervention may be necessary to remove necrotic bowel segments or to address complications [122]. For proctitis, the AGA recommends mesalamine suppositories for mild-to-moderate ulcerative proctitis due to their efficacy in inducing and maintaining remission [126]. Topical corticosteroids are indicated for patients unresponsive to mesalamine, while oral 5-aminosalicylic acid (5-ASA) agents or biologic agents, such as anti-TNF therapies, are indicated for severe cases or for patients unresponsive to topical corticosteroids [126,127].

#### 3.5.8. *Clostridioides difficile* (*C. difficile*) and Sequelae

*Clostridioides difficile* infection (CDI) is the most common healthcare-associated infection in the US, occurring after a disruption of the colonic microbiota, where *C. difficile* normally resides, typically due to recent antibiotic exposure [128,129,130,131]. Transmission occurs via the fecal–oral route, and major risk factors include recent or prolonged antibiotic use, immunodeficiency, hospitalization, advanced age, and underlying morbidities [128,131]. Clinical manifestations of CDI classically present as watery diarrhea, abdominal pain, fever, and leukocytosis, although severe cases may progress to pseudomembranous colitis, toxic megacolon, and/or death [128,132]. The prevalence of CDI in TBI patients in the ICU ranges between 0.6 and 2.6%, with a higher risk associated with prolonged antibiotic use and ICU stay, the presence of CNS devices, and the use of enteral nutrition [133,134,135]. To treat CDI, the American Society of Colon and Rectal Surgeons recommends oral vancomycin or fidaxomicin as first-line therapy and fecal microbiota transplantation for recurrent or refractory cases [128]. Prevention of CDI is dependent on antibiotic stewardship and infection control measures [132].

### 3.6. Pancreatic Disorders (Table 6)

#### Pancreatitis (Acute and Chronic) and Hyperamylasemia

Pancreatitis is an inflammatory condition of the pancreas that can cause local injury, systemic inflammatory response syndrome, and organ failure. Symptoms typically include severe epigastric or left upper quadrant pain that may radiate to the back, nausea, vomiting, and fever [136,137]. The prevalence of pancreatitis in patients with TBI is limited. However, TBI can lead to systemic inflammation and dysautonomia, which may predispose patients to various gastrointestinal conditions, including pancreatitis. A study by de Toledo et al. found that increases in pancreatic enzymes, including amylase, were observed in 57% of children with severe TBI, suggesting a potential link between TBI and pancreatic dysfunction [138]. Treatment of acute pancreatitis first involves aggressive IV fluid resuscitation, typically with lactated Ringer’s solution, to maintain adequate hydration and organ perfusion [139,140]. Opioid analgesics are also indicated for pain relief, and early enteral nutrition is preferred over parenteral nutrition to maintain gut integrity and reduce the risk of infections [139,140].

**Table 6:** Pancreatic disorders.

Disorder	Mechanisms Post-TBI	Symptoms	Treatment
Pancreatitis	Systemic inflammation Dysautonomia Stress responses leading to pancreatic dysfunction	Severe epigastric or left upper quadrant pain Nausea, vomiting Fever	IV fluid resuscitation Pain management Early enteral nutrition Antibiotics for infected necrosis
Hyperamylasemia	Systemic inflammation and stress responses leading to pancreatic dysfunction	Symptoms are related to the underlying cause (example: pancreatitis)	Treat the underlying condition Supportive care (hydration, antibiotics, etc.) Monitoring of amylase levels

### 3.7. Other Disorders (Table 7)

#### Acalculous Cholecystitis

Acalculous cholecystitis is an inflammation of the gallbladder without the presence of gallstones that is often seen in critically ill patients and is associated with factors such as fasting, parenteral nutrition, and mechanical ventilation, which can lead to ischemia of the gallbladder wall. Symptoms can include fever, right upper quadrant abdominal pain, nausea, vomiting, abdominal distension, and sepsis. The prevalence of acalculous cholecystitis in TBI patients is limited. However, TBI patients are often critically ill and may require mechanical ventilation and parenteral nutrition, which are risk factors for developing acalculous cholecystitis. A study by Mossaab et al. highlighted that acalculous cholecystitis is common in intensive care unit patients, which can include those with severe TBI [141]. For definitive treatment, early cholecystectomy, typically within 72 h, is recommended. However, for patients who are not suitable surgical candidates, percutaneous cholecystostomy can be a temporary measure used to drain the gallbladder and control infection [141,142].

**Table 7:** Other disorders.

Disorder	Mechanisms Post-TBI	Symptoms	Treatment
Acalculous Cholecystitis	Factors such as fasting, parenteral nutrition, and mechanical ventilation can lead to gallbladder ischemia	Fever, right upper quadrant pain Nausea, vomiting Signs of systemic inflammation/sepsis	Broad-spectrum antibiotics Cholecystectomy Percutaneous cholecystostomy

## 4. Conclusions

The GI system is integrally connected to the recovery of patients with TBI, given its central role in systemic inflammation, immune modulation, and nutrient absorption, all of which can significantly influence neurological outcomes. After TBI, neuroinflammatory processes and autonomic dysregulation frequently disrupt GI motility, increase intestinal permeability, and alter the gut microbiota, resulting in dysbiosis and impaired gut barrier function. These changes facilitate translocation of microbial products and proinflammatory mediators into the systemic circulation, amplifying systemic inflammation and contributing to secondary brain injury, thereby impeding neurological recovery.

GI dysfunction, however, can also occur post-TBI, given the elevated circulation of proinflammatory cytokines, impaired intestinal transit, and a shift towards pathogenic bacterial populations, which perpetuate neuroinflammation and worsen cognitive and functional outcomes. Alterations in bile acid metabolism and gut-derived metabolites further modulate both local and central immune responses, linking GI health to brain recovery and highlighting the importance of metabolic crosstalk between the gut and the CNS.

Lastly, the brain–gut axis—a bidirectional communication network involving neural, immune, and endocrine pathways—serves as the interface through which TBI-induced changes in the CNS affect GI physiology and vice versa. Disruption of this axis after TBI is now recognized as a key factor in the pathogenesis of secondary injury and represents a promising therapeutic target.

With the GI system’s significance in TBI, clinical assessment of GI dysfunction in TBI should be integrated into routine care. This includes vigilant monitoring for GI motility disorders (such as ileus or delayed gastric emptying), assessment of intestinal permeability (e.g., clinical signs of “leaky gut” or laboratory markers), and evaluation for dysbiosis (e.g., unexplained diarrhea, infection, or malabsorption). Potential biomarkers for monitoring gut health and injury progression include analysis of gut microbiota composition (via stool studies), measurement of circulating inflammatory mediators (such as TNF-α and IL-1β), and markers of intestinal barrier integrity (e.g., D-lactate and endotoxin). While these are not yet standard in all clinical settings, they are increasingly recognized as valuable adjuncts in research and specialized care [75,143,144,145]

Therapeutic strategies targeting the GI system in TBI patients should focus on both prevention and management of GI dysfunction to optimize neurological recovery and reduce secondary brain injury. Dietary interventions—including early enteral nutrition, macronutrient optimization, and specialized diets—can support gut health and modulate neuroinflammation. There is emerging evidence that specific macronutrients (such as omega-3 fatty acids and high-quality proteins) and dietary patterns may promote resilience against secondary insults and support neurological recovery [146,147].

Future directions include the need for well-designed clinical trials to establish standardized protocols for GI-targeted therapies in TBI, as current evidence is largely based on preclinical studies and small clinical trials. There is a lack of large-scale, guideline-based recommendations for the routine use of microbiome-targeted interventions in TBI, and further research is required to define optimal patient selection, timing, and specific therapeutic regimens [143,148].

In summary, incorporating systematic assessment and targeted management of the GI system can help optimize neurological recovery and reduce secondary brain injury in patients with TBI.

## Data Availability

No new data were created or analyzed in this study. Data sharing is not applicable to this article.

## Abbreviations

The following abbreviations are used in this manuscript:

5-ASA	5-Aminosalicylic Acid
5-HT	5-Hydroxytryptamine (Serotonin)
ADL	Activities of Daily Living
AGA	American Gastroenterological Association
CDI	*Clostridioides difficile* Infection
CNS	Central Nervous System
DKA	Diabetic Ketoacidosis
GERD	Gastroesophageal Reflux Disease
GCS	Glasgow Coma Scale
GI	Gastrointestinal
H2RAs	H2-Receptor Antagonists
HHS	Hyperosmolar Hyperglycemic State
HPA	Hypothalamic–Pituitary–Adrenal (Axis)
IBS	Irritable Bowel Syndrome
ICU	Intensive Care Unit
IL-1β	Interleukin-1 Beta
IV	Intravenous
MeSH	Medical Subject Headings
NPO	Nil Per Os (Nothing by Mouth)
NSAIDs	Nonsteroidal Anti-Inflammatory Drugs
PPIs	Proton Pump Inhibitors
SBO	Small Bowel Obstruction
SIBO	Small Intestinal Bacterial Overgrowth
SMA	Superior Mesenteric Artery
TBI	Traumatic Brain Injury
TNF-α	Tumor Necrosis Factor Alpha

## Appendix A

**Table A1:** List of search terms.

“Traumatic brain injury” OR “TBI” AND “gastrointestinal” OR “gut”	“Traumatic brain injury” OR “TBI” AND “Gastrointestinal dysmotility”	“Traumatic brain injury” OR “TBI” AND “Gut microbial dysbiosis”	“Traumatic brain injury” OR “TBI” AND “Malnutrition”	“Traumatic brain injury” OR “TBI” AND “Hyperphagia”
“Traumatic brain injury” OR “TBI” AND “Hyperglycemia”	“Traumatic brain injury” OR “TBI” AND “Diabetic ketoacidosis” OR “DKA”	“Traumatic brain injury” OR “TBI” AND “Hyperosmolar hyperglycemic state” OR “HHS”	“Traumatic brain injury” OR “TBI” AND “Hypoglycemia”	“Traumatic brain injury” OR “TBI” AND “Oral ulcers”
“Traumatic brain injury” OR “TBI” AND “Oral candidiasis” OR “Oral thrush”	“Traumatic brain injury” OR “TBI” AND “Herpes Simplex Virus infection” OR “Oral herpes” OR “HSV”	“Traumatic brain injury” OR “TBI” AND “Glossitis”	“Traumatic brain injury” OR “TBI” AND “Sialorrhea” OR “Hypersalivation”	“Traumatic brain injury” OR “TBI” AND “Dysphagia”
“Traumatic brain injury” OR “TBI” AND “Gastroesophageal Reflux Disease” OR “GERD”	“Traumatic brain injury” OR “TBI” AND “Esophagitis”	“Traumatic brain injury” OR “TBI” AND “Barrett’s esophagus”	“Traumatic brain injury” OR “TBI” AND “Esophageal cancer”	“Traumatic brain injury” OR “TBI” AND “Esophageal adenocarcinoma”
“Traumatic brain injury” OR “TBI” AND “Gastritis”	“Traumatic brain injury” OR “TBI” AND “Peptic Ulcer Disease” OR “PUD”	“Traumatic brain injury” OR “TBI” AND “Cushing ulcer”	“Traumatic brain injury” OR “TBI” AND “Gastric Cancer” OR “Stomach Cancer”	“Traumatic brain injury” OR “TBI” AND “Gastroparesis” OR “Delayed gastric emptying”
“Traumatic brain injury” OR “TBI” AND “Gastric Polyps”	“Traumatic brain injury” OR “TBI” AND “Recurrent Emesis”	“Traumatic brain injury” OR “TBI” AND “Duodenitis”	“Traumatic brain injury” OR “TBI” AND “Small intestinal bacterial overgrowth” OR “SIBO”	“Traumatic brain injury” OR “TBI” AND “Small bowel obstruction” OR “SBO”
“Traumatic brain injury” OR “TBI” AND “Paralytic Ileus” OR “Ileus”	“Traumatic brain injury” OR “TBI” AND “Superior Mesenteric Artery Syndrome” OR “SMAS”	“Traumatic brain injury” OR “TBI” AND “Constipation”	“Traumatic brain injury” OR “TBI” AND “Bowel incontinence” OR “Fecal incontinence”	“Traumatic brain injury” OR “TBI” AND “Incontinence-associated dermatitis”
“Traumatic brain injury” OR “TBI” AND “Incontinence” AND “Dermatitis”	“Traumatic brain injury” OR “TBI” AND “Irritable Bowel syndrome” OR “IBS”	“Traumatic brain injury” OR “TBI” AND “Mesenteric Ischemia”	“Traumatic brain injury” OR “TBI” AND “Ischemic colitis”	“Traumatic brain injury” OR “TBI” AND “Proctitis”
“Traumatic brain injury” OR “TBI” AND “*Clostridioides difficile* infection” OR “*C. diff*”	“Traumatic brain injury” OR “TBI” AND “Pseudomembranous colitis”	“Traumatic brain injury” OR “TBI” AND “Toxic megacolon”	“Traumatic brain injury” OR “TBI” AND “Pancreatitis” OR “Hyperamylasemia”	“Traumatic brain injury” OR “TBI” AND “Cholecystitis” OR “Acalculous cholecystitis”
